# Expression of Cancer‐Testis Antigens MAGE‐A1, MAGE‐A4, NY‐ESO‐1 and PRAME in Bone and Soft Tissue Sarcomas: The Experience From a Single Center in China

**DOI:** 10.1002/cam4.70750

**Published:** 2025-03-28

**Authors:** Anni Chen, Yuling Qiu, Ying‐Tzu Yen, Chun Wang, Xiaolu Wang, Chunhua Li, Zijian Wei, Lin Li, Lixia Yu, Fangcen Liu, Rutian Li

**Affiliations:** ^1^ Department of Oncology, Nanjing Drum Tower Hospital, Affiliated Hospital of Medical School, Nanjing University Nanjing China; ^2^ The Comprehensive Cancer Center, Nanjing Drum Tower Hospital, Affiliated Hospital of Medical School, Nanjing University Nanjing China; ^3^ Department of Pathology, Nanjing Drum Tower Hospital Affiliated Hospital of Medical SchoolNanjing University Nanjing China

**Keywords:** cancer testis antigen, immunotherapy, MAGE‐A4, NY‐ESO‐1, sarcoma

## Abstract

**Objective:**

Sarcomas are a heterogeneous group of malignancies, low disease‐control levels and the limited durability of responses have prompted the exploration of various novel immunotherapeutic approaches. To preliminarily explore the feasibility of cancer vaccines based on cancer testis antigen in the immunotherapy of sarcomas, we investigate the expression of Cancer/Testis Antigens (CTA) MAGE‐A4, PRAME, MAGE‐A1, KK‐LC‐1, and NY‐ESO‐1 in bone and soft tissue sarcomas, with the aim of assessing their potential for use in sarcoma immunotherapy and determining their expression levels in different subtypes.

**Methods and Results:**

We employed immunohistochemistry and multiplex immunostaining microarrays (MI chips) to assess the expression of MAGE‐A4, PRAME, MAGE‐A1, KK‐LC‐1, and NY‐ESO‐1 in 21 cases of undifferentiated pleomorphic sarcoma (UPS), 26 cases of smooth muscle sarcoma, 28 cases of liposarcoma, 40 cases of osteosarcoma (OS), and 13 cases of chondrosarcoma. MAGE‐A1 showed the highest expression in osteosarcoma (32.50%), while it was lower in liposarcoma and undifferentiated pleomorphic sarcoma (10.71% and 10.00%) and undetectable in chondrosarcoma. MAGE‐A4 expression was elevated in osteosarcoma and undifferentiated pleomorphic sarcoma (40.00% and 33.00%), but lower in liposarcoma and smooth muscle sarcoma (17.00% and 33.00%). NY‐ESO‐1 expression was relatively low across all sarcoma subtypes. PRAME expression was highest in undifferentiated pleomorphic sarcoma (47.62%) and low in chondrosarcoma (7.69%). None of the sarcomas expressed KK‐LC‐1. Additionally, while there was no statistically significant correlation between CTA expression and patient age or gender, some differences related to age and gender were observed.

**Conclusions:**

CTA expression in bone and soft tissue sarcomas was correlated with both CTA type and sarcoma subtype, showing relatively high levels of expression in undifferentiated pleomorphic sarcoma (UPS) and osteosarcoma (OS). The poly‐expression of MAGE‐A4, PRAME, and MAGE‐A1 across all subtypes suggests that these antigens may serve as potential targets for sarcoma‐specific immunotherapy.

## 1 Introduction

Sarcomas, including bone sarcomas and Soft‐tissue sarcomas (STS), refer to a group of rare mesenchymal tumors that account for around 1% of adult malignant tumors with a high recurrence rate [1, 2]. According to the 2020 World Health Organization (WHO) classification of tumors of soft tissue and bone, there are quite a few different histologic subtypes of sarcomas with remarkable genetic and pathological heterogeneity [3]. Bone sarcomas include osteosarcoma, Ewing sarcoma, and chondrosarcoma [4]. Among all adult STS, leiomyosarcoma (LMS), liposarcoma(LPS) and undifferentiated pleomorphic sarcoma (UPS) are the most common [5]. Surgery is the primary treatment for sarcomas [5], but even after combined radiotherapy and chemotherapy, the long‐term survival rate of patients with STS is still not optimistic [6, 7]; the 5‐year survival rate for sarcomas with distant metastasis is only 52% [8]. There have also been many other attempts at the treatment of STS. In recent years, some controversy exists surrounding the superiority of neoadjuvant chemotherapy (NCT) [1, 9, 10]; immunotherapy may be a way out. Notably, a Phase II study evaluating the safety and efficacy of CMB305 (NY‐ESO‐1 pulsed DC vaccination) in combination with atezolizumab compared to atezolizumab alone in STS demonstrated the benefits of combination immunotherapy [11]. Although CMB305 in combination with atezolizumab did not significantly improve progression‐free survival (PFS) or overall survival (OS) compared to atezolizumab alone, it may induce higher rates of NY‐ESO‐1‐specific T‐cell and antibody responses. In addition, patients who develop anti‐NY‐ESO‐1 T‐cell responses appear to have a longer OS [11]. However, not all subtypes benefit from this treatment. In addition to individual subtypes, many clinical trials have shown that immune checkpoint inhibitors (ICIs) alone do not benefit patients with sarcoma [12, 13, 14]. Combined with other kinds of ICIs [15] or combined with other treatments [16, 17], ICIs may provide new potential. In a clinical trial of patients with locally advanced, unresectable, or metastatic sarcoma, nivolumab(a monoclonal antibody against programmed death 1/PD‐1) achieved only a 5% response rate, whereas the combination of nivolumab and ipilimumab (a monoclonal antibody against T‐lymphocyte‐associated protein 4/CTLA‐4) achieved a 16% response rate [15]. On the other hand, changes in the characteristics of cellular infiltration within the tumor microenvironment have been observed following neoadjuvant immunotherapy, including increases in CD4^+^ T cells and B cells, both of which suggest the potential for an enhanced immune response [18]. Furthermore, the tumor immune microenvironment (TiME) of sarcomas is highly heterogeneous, reflecting their diverse histological characteristics, and targeting the TiME may have clinical implications and potential therapeutic effects [18]. Despite the positive results associated with neoadjuvant immunotherapy, several challenges remain, such as the risk of hyperprogressive disease (HPD) and limitations related to specific subtypes [19]. These factors suggest that the use of immunotherapy in sarcoma is still at an exploratory stage. Nevertheless, with further investigation of the tumor immune microenvironment and the selection of appropriate biomarkers, there is potential to improve therapeutic efficacy. To benefit more patients with sarcoma, further research and exploration of immunotherapy are required.

Cancer immunotherapy involves non‐specific immunotherapy, tumor vaccines, adoptive cell transfer (ACT) and monoclonal antibody‐based treatment, among which ACT and vaccines have been investigated as emerging immunotherapies in the field of sarcoma [20, 21, 22]. Genetically engineered T‐cell receptors (TCRs) and chimeric antigen receptor (CAR) T cells, as representatives of ACT, achieve killing effects by increasing the specificity and responsiveness of T cells to tumor cells [23, 24]. The implementation of all these therapies depends on the choices of applicable targets or antigens. Cancer/Testis Antigens (CTA) are a group of tumor‐associated antigens (TAA) expressed in the testis or tumor tissues from different origins, which can be detected in relevant tissues through immunohistochemistry or in serum by specific serological reagents [25]. Antigens derived from CTAs can be recognized by T lymphocytes and are therefore capable of leading to a potent antitumor immune response [26]. CTAs have the following advantages that make them important targets for immunotherapy. First of all, as a marker of quite a few types of tumors, CTAs avoid the individualized requirements of neoantigens and the limitation of immune recognition while retaining tumor specificity and immunogenicity. Secondly, CTAs can be detected directly in a short period without the need for genetic testing. Thirdly, CTAs maintain both peculiarity and universality. With certain tumor specificity and immunogenicity, CTAs can be used in a variety of immunotherapy methods, including tumor vaccines and TCR‐T therapies. There have been advances in novel immunotherapies targeting CTAs in the field of synovial sarcoma [27]. Vaccines targeting New York esophageal squamous cell carcinoma 1 (NYESO‐1) [28, 29] and TCR‐T therapies targeting NYESO‐1 [30] or melanoma‐associated antigen A4 (MAGE‐A4) [31] are reported under clinical trials, making CTAs an attractive therapeutic target for sarcomas.

Lung, ovarian, bladder, melanoma, and breast tumors had a large proportion of CTA expression [32]. It has been reported that several types of STS highly express CTAs, and MAGE, NY‐ESO‐1, and preferentially expressed antigen of melanoma (PRAME) are the most commonly expressed and studied CTAs in sarcomas [26]. NY‐ESO‐1 is the most notable one of them, which is highly expressed in most synovial sarcomas (SSs) [33] and myxoid/round cell liposarcomas (MRCLs) [34]. Osteosarcoma, synovial sarcoma, and myxoid/round cell liposarcoma show very high expression of MAGE and PRAME [26]. In China, the crude incidence rate of STS is 2.91/100000 with about 40,000 new cases each year [35]. Nevertheless, there is scarcely any report on the expression of CTAs in Chinese sarcoma patients, and there is also a lack of data on many other sarcoma types.

Immunohistochemistry (IHC) is a valuable tool in the diagnosis of sarcoma. Multiplex immunostaining chip (MI chip) is a novel technology which can examine multiple antigens (Ags) in the same tissue section [36, 37]. The MI chip used in microarrays allows simultaneous detection of different analytes on a single chip, outperforming traditional laboratory assays.

Thus, we aim to investigate the expression of MAGE‐A4, PRAME, MAGE‐A1, Kita‐Kyushu lung cancer antigen‐1 (KK‐LC‐1) and NY‐ESO‐1 in osteosarcoma (OS), liposarcoma (LPS), leiomyosarcoma (LMS), chondrosarcoma (CS) and undifferentiated pleomorphic sarcoma (UPS) by immunohistochemistry and MI chip to provide a basis for novel immunotherapy of sarcomas.

## 2 Materials and Methodsnovel

### 2.1 Patients and Tissue Specimens

A retrospective collection of 128 patients with bone and soft tissue sarcoma who retained tissue specimens in Nanjing Drum Tower Hospital from 2014 to 2021. There were 39 cases of osteosarcoma, 28 cases of liposarcoma, 26 cases of leiomyosarcoma, 22 cases of UPS and 13 cases of chondrosarcoma evaluated. All tissue samples were confirmed as sarcoma by two pathologists, and all were available for immunohistochemical testing. Immunohistochemistry was used to detect the expression of MAGE‐A4, PRAME, MAGE‐A1, KK‐LC‐1 and NY‐ESO‐1. The immunohistochemical results were observed by two pathologists according to the 2017 International Union Against Cancer (UICC)/American Joint Commission on cancer (AJCC) staging standard (the eighth Edition).

### 2.2 Immunohistochemical Staining and Evaluation

The IHC procedure was performed with reference to a previous study of our team [38]. Paraffin sections of sarcoma specimens were routinely dewaxed and hydrated with xylene and gradient alcohol after 30 min in a dryer that keeps 90°C; Antigen repair: stand in 99°C citrate buffer for 20 min, cool to room temperature, soak in dH_2_O for 5 min, repeat 3 times, and soak in PBS for 5 min, repeat 3 times; Dry the slices, incubate them with 3% H_2_O_2_ for 15 min (extinguishing endogenous peroxidase), repeat the above cleaning process of dH_2_O and PBS, and use BSA sealing solution for 30 min; The primary antibody of mice was incubated at 37°C for 1–2 h or 4°C overnight, soaked in PBS for 5 min and repeated 3 times; The secondary antibodies including anti‐MAGE‐A4 antibody (mouse monoclonal antibody, OTI1F9, Abcam, USA), anti‐PRAME anti body (rabbit monoclonal antibody, EPR20330, Abcam, USA), anti‐MAGE‐A1 antibody (mouse monoclonal antibody, MA454, Thermo Fisher Scientific), anti‐KK‐LC‐1 antibody (mouse monoclonal antibody, CL4762, Thermo Fisher, USA), and anti‐NY‐ESO‐1 antibody (mouse monoclonal antibody, E978, Santa Cruz Biotechnology), were incubated for 15 min, soaked in PBS for 5 min, repeated 3 times, and stained with DAB chromogenic solution for 2–10 min, Soak in dH_2_O for 5 min, repeat 3 times, dye in hematoxylin for 1–2 min, rinse with running water, dyed in eosin for 1–3 min, soak in 75% ethanol for 1 min, 85% ethanol for 1 min, 95% ethanol for 2 min, absolute ethanol for 2 min, xylene I for 3 min, xylene II for 3 min, sealed with neutral gum, observed, and took photos under the microscope.

All staining results were obtained by two experienced pathologists reading the films under the microscope and evaluated with the staining intensity combined with the number of positive cells. Cell color rendering intensity: 0 point for no‐color rendering, 1 point for weak brownish yellow, 2 points for medium strong brownish yellow, and 3 points for strong brownish brown; Proportion of positive cells: 0 for negative, 1 for ≤ 10%, 2 for 11% ~ 50%, 3 for 51% ~ 75% and 4 for > 75%. When the two scores are multiplied, 0 is defined as negative, 1–3 is defined as weak positive, 4–7 is defined as medium positive, and 8–12 is defined as strong positive.

### 2.3 Data Sources for Comparison

We searched PubMed and CTDatabase [39] from inception to July 1, 2022, without any restrictions. Search terms included those related to sarcoma, CTA, and their variants. We found representative reviews with detailed data as references, which were compared with our results in the subsequent discussion section.

### 2.4 Statistical Analysis

The *χ* ^2^ test or Fisher exact test was used to evaluate the association between variables. A two‐sided *p* value less than 0.05 was considered significant. The data analyses were performed using the SPSS Statistics version 26 (IBM China Ltd., Shanghai, China). *p* < 0.05 was considered statistically significant.

## 3 Results

### 3.1 Patient and Clinicopathological Parameters

The basic data (sex and age) and clinicopathological data (location, diameter, treatment and WHO/FNCLCC grading) for 128 sarcomas are shown in Table 1, including osteosarcoma(*n* = 40), liposarcoma(*n* = 28), leiomyosarcoma(*n* = 26), UPS(*n* = 21) and chondrosarcoma(*n* = 13). Among all these sarcomas, the age of osteosarcoma patients is the youngest (median = 25.5, average = 32.13), while UPS patients are relatively older than others (median = 63, average = 61.45). Leiomyosarcoma and chondrosarcoma have an obvious gender preference. The number of women with leiomyosarcoma is significantly larger than that of men (female *n* = 22, male *n* = 4), while the number of men with chondrosarcoma is twice as large as that of women (male *n* = 8, female *n* = 4). In terms of size, liposarcoma samples had the largest mean diameter (17.91 cm), while UPS samples had the smallest mean diameter (6.55 cm). Samples of osteosarcoma (77.5%), chondrosarcoma (76.92%), and UPS (47.62%) were mainly from proximal, and liposarcomas (71.43%) were mainly from abdominal organs or the retroperitoneum. Most patients with osteosarcoma received chemotherapy before surgery (55%), and necrosis after chemotherapy was observed.

**TABLE 1:** Patient and clinicopathological parameters.

Diagonoses	Osteosarcoma		Liposarcoma		Leiomyosarcoma		UPS		Chondrosarcoma		Total	
	*N*	%	*N*	%	*N*	%	*N*	%	*N*	%	*N*	%
Sex												
Male	21	52.50%	11	39.29%	4	15.38%	11	52.38%	8	61.54%	55	42.97%
Female	19	47.50%	17	60.71%	22	84.62%	9	42.86%	4	30.77%	71	55.47%
N/A	0	0.00%	0	0.00%	0	0.00%	1	4.76%	1	7.69%	2	1.56%
Age												
< 18 year	13	32.50%	0	0.00%	0	0.00%	0	0.00%	0	0.00%	13	10.16%
≥ 18 year and < 65 year	24	60.00%	22	78.57%	19	73.08%	13	61.90%	9	69.23%	87	67.97%
≥ 65 year	3	7.50%	6	21.43%	7	26.92%	7	33.33%	3	23.08%	26	20.31%
N/A	0	0.00%	0	0.00%	0	0.00%	1	4.76%	1	7.69%	2	1.56%
Median	25.5		58		55.5		63		49		52	
Mean	32.13		57.57		58.46		61.45		50.08		49.58	
Diameter (cm)												
T1	7	17.50%	1	3.57%	6	23.08%	9	42.86%	4	30.77%	27	21.09%
T2	13	32.50%	7	25.00%	11	42.31%	9	42.86%	4	30.77%	44	34.38%
T3	13	32.50%	5	17.86%	6	23.08%	1	4.76%	3	23.08%	28	21.88%
T4	5	12.50%	14	50.00%	3	11.54%	1	4.76%	1	7.69%	24	18.75%
N/A	2	5.00%	1	3.57%	0	0.00%	1	4.76%	1	7.69%	5	3.91%
Mean	10.70		17.91		9.33		6.55		8.75		11.13	
Location												
Distal extremities	7	17.50%	1	3.57%	1	3.85%	4	19.05%	1	7.69%	14	10.94%
Proximal extremities	31	77.50%	3	10.71%	1	3.85%	10	47.62%	10	76.92%	55	42.97%
Abdominal visc‐era and retroperit‐oneal	0	0.00%	20	71.43%	11	42.31%	1	4.76%	0	0.00%	32	25.00%
Trunk	1	2.50%	2	7.14%	0	0.00%	3	14.29%	1	7.69%	7	5.47%
Pelvic part	0	0.00%	1	3.57%	11	42.31%	1	4.76%	0	0.00%	13	10.16%
Other	1	2.50%	1	3.57%	2	7.69%	0	0.00%	0	0.00%	4	3.13%
N/A	0	0.00%	0	0.00%	0	0.00%	2	9.52%	1	7.69%	3	2.34%
Necrosis												
< 50%	8	20.00%										
≥ 50%	15	37.50%										
N/A	17	42.50%										
Treatment												
Chemotherapy	22	55.00%										
N/A	18	45.00%										
WHO/FNCLCC												
I			1	3.57%	0	0.00%	0	0.00%	6	46.15%	7	5.47%
II			3	10.71%	1	3.85%	1	4.76%	3	23.08%	8	6.25%
I ~ II			1	3.57%	0	0.00%	0	0.00%	1	7.69%	2	1.56%
III			0	0.00%	0	0.00%	8	38.10%	0	0.00%	8	6.25%
N/A			23	82.14%	25	96.15%	12	57.14%	3	23.08%	63	49.22%
Total	40		28		26		21		13		128	

*Note:* T1, ≤ 5 cm; T2, > 5 and ≤ 10 cm; T3, > 10 and ≤ 15 cm; T4, > 15 cm.

Abbreviation: N/A, none or not available.

### 3.2 The Results of Immunohistochemical Staining

**FIGURE 1:**
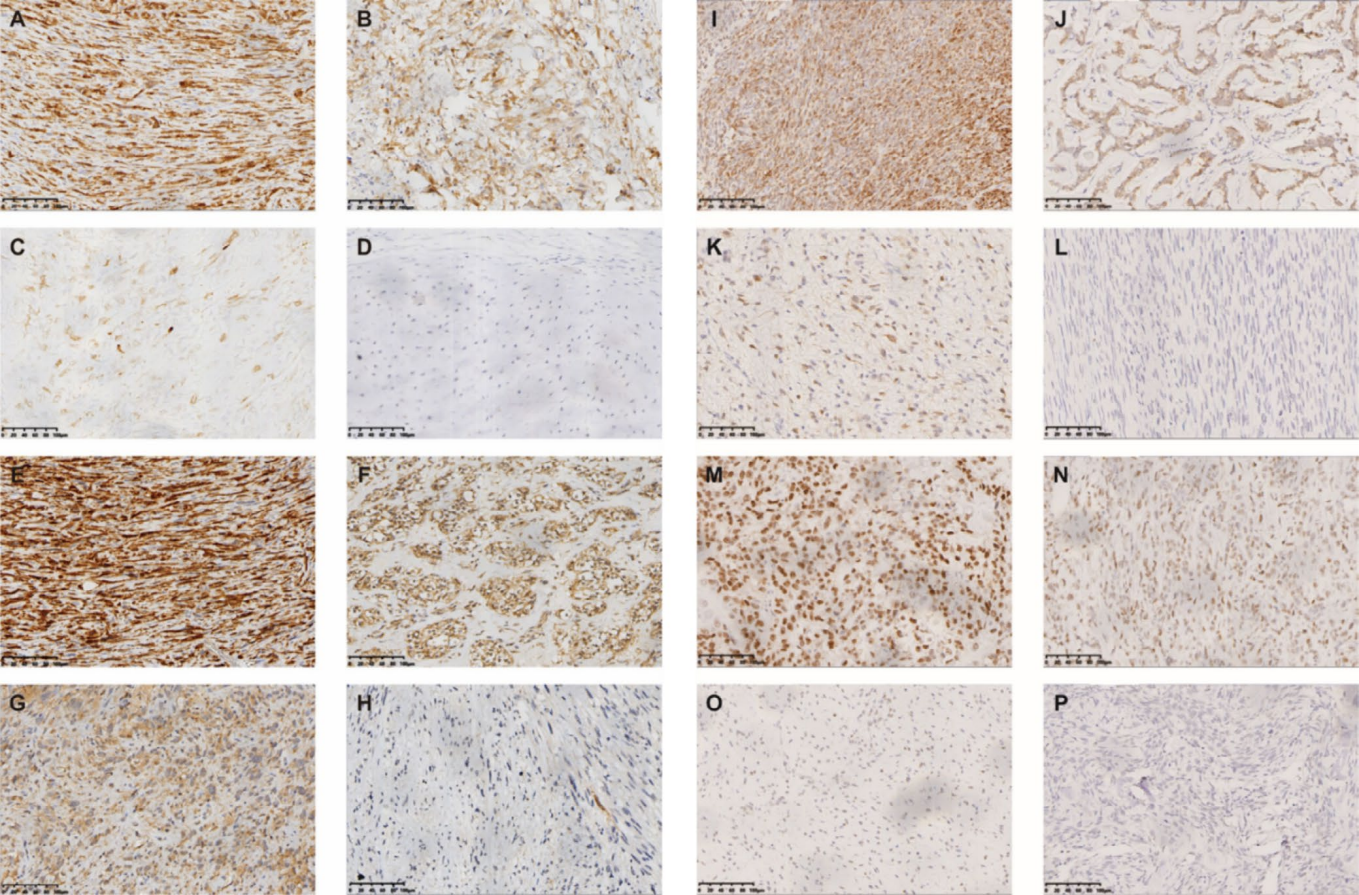
Microscopic immunochemistry results presenting the expression of MAGE‐A1, MAGE‐A4, NY‐ESO‐1, and PRAME. (A–D) The sample picture of strong positive (A, +++/++++), medium positive (B, ++), weak positive (C, +) and negative (D) of MAGE‐A1. (E–H) The sample picture of strong positive (E, +++/++++), medium positive (F, ++), weak positive (G, +) and negative (H) of MAGE‐A4. (I–L) The sample picture of strong positive (I, +++/++++), medium positive (J, ++), weak positive (K, +) and negative (L) of NY‐ESO‐1. (M–P) The sample picture of strong positive (M, +++/++++), medium positive (N, ++), weak positive (O, +) and negative (P) of PRAME.

In most samples, the staining was not homogenous (Table 2). Osteosarcoma samples were most likely to strongly express CTAs (35%), while only 7.69% of leiomyosarcoma samples had 3+ or 4+ staining. Among all CTAs tested, MAGE‐A4 had the largest number of positive samples, and MAGE‐A1 was most intended to be strongly expressed (Table S1). From the perspective of sarcoma type, the CTA positive rate in chondrosarcoma samples was the lowest, and the positive rate in UPS samples was the highest (Table S2). There are 1 osteosarcoma sample and 2 liposarcoma samples expressing four CTAs (Table S2). Figure 1 shows the immunohistochemical results of different degrees of staining.

**TABLE 2:** immunohistochemical results of staining intensity.

Diagnosis	Total	+		++		+++/++++	
	*N*	*N*	%	*N*	%	*N*	%
Osteosarcoma	40	13	32.50%	9	22.50%	14	35.00%
Liposarcoma	28	5	17.86%	2	7.14%	8	28.57%
Leiomyosarcoma	26	6	23.08%	6	23.08%	2	7.69%
UPS	21	13	61.90%	7	33.33%	5	23.81%
Chondrosarcoma	13	1	7.69%	—	—	—	—
Total	128	38	29.69%	24	18.75%	29	22.66%

Abbreviation: *N*, number.

### 3.3 Statistical Analysis

The immunohistochemical expressions of CTAs were not correlated with sex, age, or diameter statistically(*p* value ≥ 0.05) in osteosarcoma, liposarcoma, leiomyosarcoma, UPS, and chondrosarcoma (Table 3). However, all samples of osteosarcoma patients aged 65 or older expressed MAGE‐A4 (Table S3). In liposarcoma specimens, those from males are quite more likely to express CTAs than females, among which 3 specimens expressed MAGE‐A1, MAGE‐A4, and NY‐ESO‐1 at the same time (Tables S3 and S4).

**TABLE 3:** The results of *χ* ^2^ test or Fisher exact test.

Diagnosis	Variables	Immunohistochemical results: P				Total
		MAGE‐A1	MAGE‐A4	NY‐ESO‐1	PRAME	
Osteosarcoma	Sex	0.906	0.366	—	1.000	0.775
	Age	0.508	0.061	—	0.196	0.250
	Diameter	0.591	0.452	—	0.326	0.603
Liposarcoma	Sex	0.050	0.062	0.050	1.000	0.381
	Age^a^	0.107	0.285	0.285	0.191	0.622
	Diameter	1.000	0.724	1.000	0.724	0.566
Leiomyosarcoma	Sex	0.511	1.000	—	1.000	0.591
	Age^a^	1.000	1.000	—	1.000	0.661
	Diameter	0.391	1.000	—	0.697	0.408
UPS	Sex	0.336	1.000	1.000	0.406	1.000
	Age^a^	1.000	0.613	1.000	1.000	1.000
	Diameter	0.584	1.000	1.000	0.874	1.000
Chondrosarcoma	Sex	—	—	—	—	—
	Age	—	—	—	—	—
	Diameter	—	—	—	—	—

^a^ Comparison of ≥ 18 year and < 65 year and ≥ 65 year.

### 3.4 The Results of MI Chip and Comparism

All of these sarcomas did not express KK‐LC‐1; the positive rates of other CTAs are as follows (Table 4). The probability of PRAME expression was the highest (21.88%) in all the sarcoma samples we examined. Among these CTAs, osteosarcomas tended to express MAGE‐A4 (40%), and leiomyosarcomas (26.92%) and UPS (47.62%) tended to express PRAME, while there was no significant difference in the expression rates of these four CTAs in liposarcoma. Only one case of chondrosarcoma expressed PRAME (Table S4, 7.69%), and the other CTAs were not detected in any samples of chondrosarcoma. The expression rate of MAGE‐A1 in osteosarcoma was the largest (32.5%), and that in liposarcoma was the smallest (10.71%). However, MAGE‐A4 expression has been reported only in liposarcoma [44] and leiomyosarcoma [51], and the expression in liposarcoma was similar to that in our study. The expression of MAGE‐A4 and PRAME in these sarcomas was significantly different from that in references. Also, the expression rate of NY‐ESO‐1 in our assay results was also much lower than that reported before, with the highest rate in liposarcoma (10.7%).

**TABLE 4:** Positive rates of MAGE‐A1, MAGE‐A4, NY‐ESO‐1, and PRAME in osteosarcoma, liposarcoma, leiomyosarcoma, UPS, and chondrosarcoma in references and our data.

Diagnosis	MAGE‐A1		MAGE‐A4		NY‐ESO‐1		PRAME	
	PubMed	DTH	PubMed	DTH	PubMed	DTH	PubMed	DTH
Osteosarcoma	—	32.50%	43.8% [40], 100% [41]	40.00%	33.3% [41], 88.89% [42]	2.50%	70% [43]	15.00%
Liposarcoma	11% [44]	10.71%	0% [45], 8.7% ~ 14.3% [40], 67.7% [40]	17.85%	55.6% ~ 100% [34, 40, 45, 46, 47, 48, 49]	10.71%	76% [50], 90% [49], 100% [44]	14.28%
Leiomyosarcoma	31% [51]	15.38%	9.1% [45], 34% [51]	11.54%	0% [52], 2% [53], 9.1% [45]	0%	6% [53]	26.92%
UPS	—	28.57%	0% [45], 60% [54]	33.33%	0% [45], 2% [53], 50% [54]	9.52%	7% [53]	47.62%
Chondrosarcoma	—	0%	21.4% [40]	0%	0% [46], 28% [48], 36% [55]	0%	0% [55]	7.69%
Total		20.31%		24.22%		4.69%		21.88%

Abbreviations: DTH, Drum Tower Hospital; UPS, undifferentiated pleomorphic sarcoma.

## 4 Discussion

It has been demonstrated that CTAs are expressed in a variety of human cancer tissues, and at least 19 CTAs have been found to elicit humoral and/or cellular immune responses in cancer patients [56]. All CTAs that we examined were among them. MAGE‐A1 and MAGE‐A4 are both members of the Melanoma Antigen Gene (MAGE) family, which are expressed in a variety of malignancies, including lung (non‐small cell), breast, colon, and ovarian cancer [57]. Preferentially expressed antigen in melanoma (PRAME) is frequently expressed in numerous solid tumors and hematological malignancies [58]. NY‐ESO‐1, being the most immunogenic among CTAs, with a variety of immunotherapies against NY‐ESO‐1 in clinical trials, including tumor vaccines and adoptive T cell therapy [59]. Kita‐Kyushu lung cancer antigen‐1 (KK‐LC‐1), also known as CT83 or cxorf61, is highly expressed in lung cancer, gastric cancer, and breast cancer, and its role in immunotherapy needs to be further explored [60].

**FIGURE 2:**
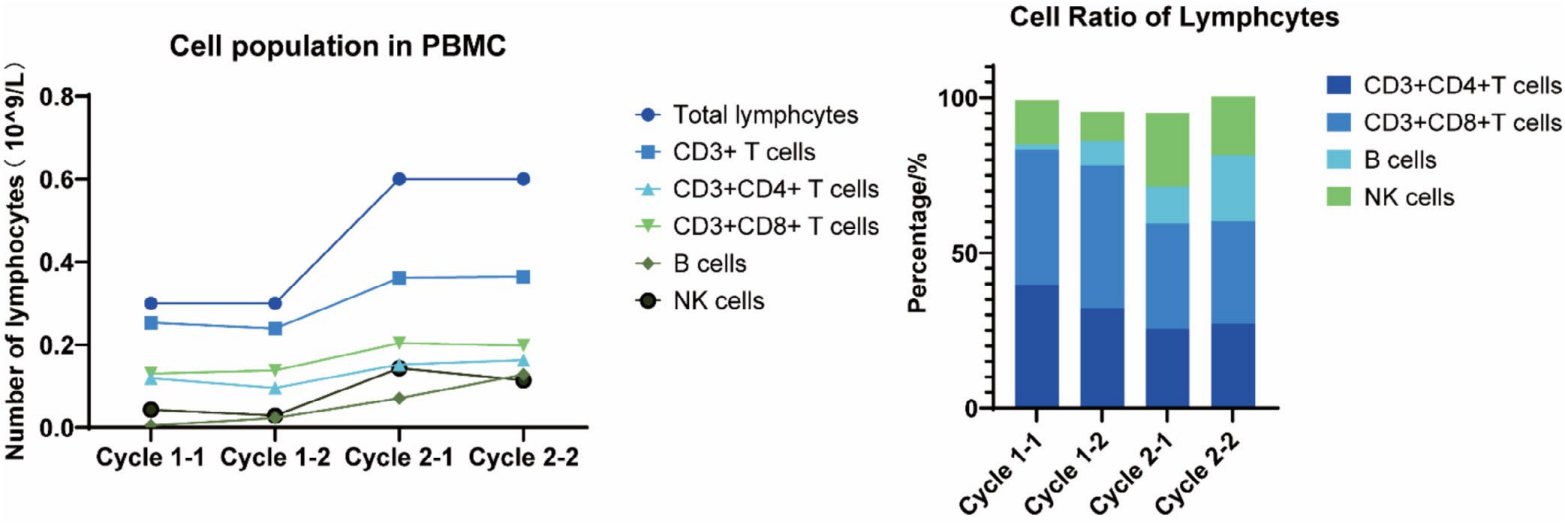
Cellular immune response after treatment. Lymphocytes, CD3^+^, CD8^+^ cytotoxic T lymphocytes, NK cells, and B lymphocytes were increased, suggesting the possibility of immune activation.

**FIGURE 3:**
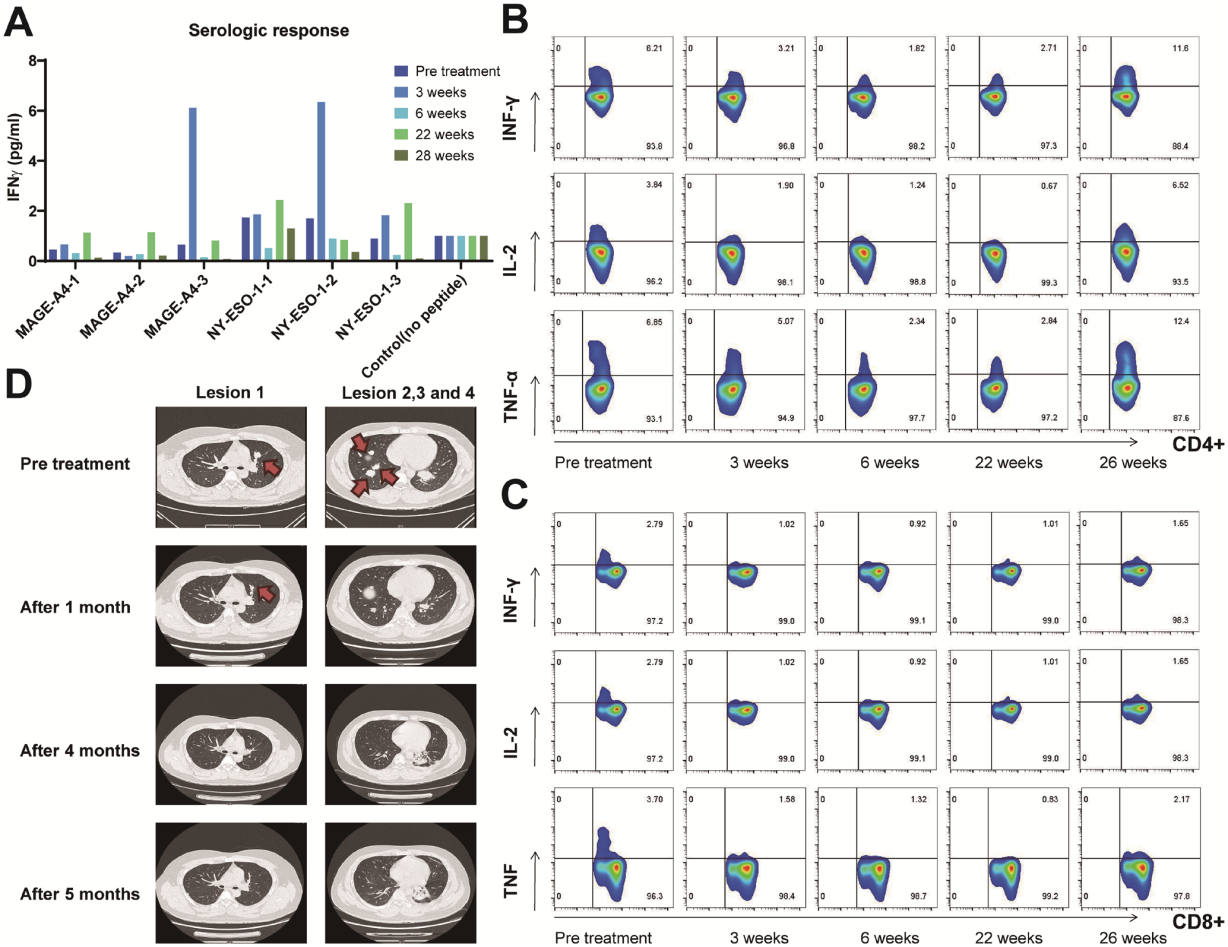
Immunological and clinical response to carcinoma‐testis antigen vaccine in patients with soft tissue sarcoma. (A) Following the vaccination, cytometry microsphere array assays demonstrated that peripheral blood mononuclear cells secreted INF‐γ levels during different phases of immune therapy. (B) Percentage of INF‐γ‐positive cells, IL‐2‐positive cells, and TNF‐α‐positive cells in CD4^+^ T cells. (C) Percentage of INF‐γ positive cells, IL‐2 positive cells, and TNF‐α positive cells in CD8^+^ T cells. (D) Representative imaging profiles of patients pre and post immunotherapy.

All of the CTAs detected have been identified as targets for cancer immunotherapy in solid cancer like breast cancer [61, 62] and so on. Cancer/Testis Antigens are a promising class of immunotherapy targets in the field of sarcoma treatment, which have shown some potential both in references [27, 28, 29, 30, 31] and in the research of our group. NY‐ESO‐1 and MAGE antigens are the most commonly targeted CTAs in sarcoma [63] and some progress has been made in modified T‐cell therapies [64]. The clinical trial carried out in the cancer center of our hospital using CTAs as tumor vaccine antigens has achieved good results. A patient with advanced fibrosarcoma who received an individualized peptide vaccine in the comprehensive tumor center of Drum Tower Hospital Affiliated to Nanjing University was analyzed. The CTA test results of the patient showed that MAGE‐A4 (+++) and NY‐ESO‐1 (++). Since April 20, 2021, the patient has completed two rounds of vaccine injection, nine times per round, and the lymphocyte subsets in each round have been tested (Figure 2). Lymphocytes, B lymphocytes, and NK cells increased significantly during the two rounds of vaccine infusion, suggesting the possibility of immune activation, and no obvious adverse reactions were observed during the period. In addition, we assayed the immunogenicity of candidate antigenic peptide vaccines via cytometry microsphere array and intracellular cytokine staining for patients across a series of time points pre‐ and post‐vaccination. Peripheral blood samples were collected at various time points post‐vaccination, and peripheral blood mononuclear cells (PBMCs) were isolated and stimulated with different antigenic peptides. Subsequently, the level of IFN‐γ expression in the supernatant was quantified by ELISPOT assay. The secretion levels of IFN‐γ in the culture supernatant showed dynamic changes, and the immune response to each peptide was observed approximately 3 weeks post‐vaccination. Among them, 2 antigenic peptides tended towards increasing at the third week (Figure 3A). And the percentage of IFN‐γ‐producing, IL‐2‐producing, and TNF‐α‐producing CD4^+^ T cells appeared to have an increasing trend pre‐ and post‐immunotherapy. At the same time, the percentage of IFN‐γ‐producing, IL‐2‐producing, and TNF‐α‐producing CD8^+^ T cells generated decreased slightly (Figure 3B,C). Following vaccination for approximately 3 weeks, the chest CT scan displayed a significant reduction of the multiple metastatic lesions in the pulmonary region (Figure 3B). Such results indicate that patients with soft tissue sarcoma who explicitly express CTAs may benefit from immunotherapy and warrant further investigation.

According to our results, the proportion of CTAs expression in UPS was the highest (76%), and PRAME was the predominant one (47.62%), suggesting that PRAME may be a therapeutic target for UPS. Among all CTAs, MAGE‐A4 has the highest expression ratio, suggesting that it is an ideal target for sarcoma immunotherapy. Also, the expression rate of MAGE‐A4 was the closest to that reported in the literature. At present, NY‐ESO‐1 is the main target of sarcoma immunotherapies in CTAs [30, 47, 59, 65, 66, 67, 68], while there are few studies on MAGE‐A4 [27, 64]. Our study demonstrated that MAGE‐A4 is not inferior to NY‐ESO‐1 in future development. It is worth mentioning that MAGE‐A4 also has the highest expression rate of CTAs expressed in osteosarcoma (40.00%), suggesting that osteosarcoma is an optimal sarcoma for the study of MAGE‐A4. Furthermore, as can be seen from our pictures (Figure 1), MAGE‐A4 showed relatively deep staining, which probably indicated a stronger antigenicity or immunogenicity and may have provided another basis for its suitability for further study. The proportion of CTAs expression in chondrosarcoma was the lowest (8%), suggesting that CTAs targeted immunotherapy may not be suitable for general application in chondrosarcoma patients. We have not detected the expression of KK‐LC‐1 in sarcomas, nor has it been reported in the relevant literature. In addition, the expression of MAGE‐A1 in osteosarcoma, UPS, and chondrosarcoma has not been reported in the literature, and our study has certain groundbreaking and reference value.

Our results are not exactly the same as those reported in the literature, and the expression rates we detected were lower than many reported in the literature. The incidence of sarcomas is low, and the sample size of some referenced studies was insufficient [41, 42, 54, 55], which may lead to poor validity and reference value. Some of our conclusions were similar in studies of similar sample sizes [44, 46]. Many literatures combine the results of DNA detection [42, 44], RNA detection [43] and western blot (WB) [49] in the laboratory. However, it seems that the clinical requirements for convenience and rapidity are not met. IHC has the highest utilization and feasibility in the diagnosis and treatment process, making its results the most clinically meaningful. In addition, regional or ethnic differences and the use of fresh tissue testing [43] may also be sources of discrepancy generation. The references cited were mainly from the USA and Japan (Table S5), while we creatively reported the expression in Chinese patients, suggesting that region may have an impact on the expression rate of CTAS in bone and soft tissue sarcomas.

In our study, there was no statistically significant correlation between CTA expression and the age or gender of patients. Nevertheless, there is some age or gender bias in the expression of CTAs in different types of sarcoma. Women have a higher incidence of liposarcoma than men, but in our sample, men are more likely to express CTAs. This suggests that male patients with liposarcoma are more suitable subjects for the study of CTAs‐targeted immunotherapy in sarcomas. In osteosarcoma, all the samples from elderly patients (age ≥ 65 years) have CTA expression, which is also worthy of further exploration and research. Insufficient sample size may be the main reason for the inability to find statistical associations, and subsequent studies with larger sample sizes are needed to explore the relationship between gender, age, and CTA expression.

## 5 Conculsion

In conclusion, we report MAGE‐A1, MAGE‐A4, NY‐ESO‐1, PRAME, and KK‐LC‐1 expression within osteosarcoma, liposarcoma, leiomyosarcoma, chondrosarcoma, and undifferentiated pleomorphic sarcoma using immunohistochemistry and MI chip. The present finding is that MAGE‐A1, MAGE‐A4, and PRAME are frequently expressed. Of these, MAGE‐A4 and PRAME exhibit strong and homogeneous expression within OS and UPS. As a highly specific and immunogenic tumor‐associated antigen expressed in malignant tumors, CTA can stimulate and induce the immune response of related lymphocytes, so as to achieve the purpose of anti‐tumors. CTA is highly expressed in soft tissue sarcomas, which is consistent with our results, so it can be used as a potential immunotherapeutic target for the treatment of sarcoma. According to the reports of relevant clinical trials and the clinical trials related to cancer testis carried out by the tumor center of our hospital, tumor vaccines and TCR‐T cell reinfusion with CTA as an immune target may be the most promising treatment for sarcomas that fail to receive multi‐line treatment, advanced, or recurrent. This study also found that the expression of CTA in various subtypes of sarcoma was different, and there were significant differences with the reports in the literature, suggesting that the expression of CTA may be related to the differences in region and sarcoma subtypes. This also requires us to expand the samples and improve the CTA detection of sarcoma for further verification. As there are differences in the expression of CTA in sarcoma subtypes, we expect to find tumor antigens with more clinical therapeutic significance in the future or combine CTA‐targeted immunotherapy with other effective treatments (such as radiotherapy, immune checkpoint inhibitors, etc.).

## Author Contributions

**Anni Chen:** data curation (lead), writing – original draft (lead), writing – review and editing (equal). **Yuling Qiu:** formal analysis (lead), visualization (lead), writing – original draft (equal), writing – review and editing (lead). **Ying‐Tzu Yen:** data curation (equal). **Chun Wang:** data curation (equal). **Xiaolu Wang:** data curation (equal). **Chunhua Li:** data curation (equal). **Zijian Wei:** data curation (equal). **Lin Li:** methodology (equal). **Lixia Yu:** methodology (equal). **Fangcen Liu:** conceptualization (equal), funding acquisition (equal), methodology (equal), project administration (equal). **Rutian Li:** conceptualization (lead), funding acquisition (equal), project administration (equal).

## Ethics Statement

The study was performed in accordance with the principles and guidelines of the Declaration of Helsinki and approved by the Ethics Committee of the Nanjing Drum Tower Hospital (2022‐052‐01). All patients provided written informed consent.

## Consent

The authors have nothing to report.

## Conflicts of Interest

The authors declare no conflicts of interest.

## Supporting information


Table S1.

## Data Availability

All data included in this study are available upon request by contacting the corresponding author.
