# Correction to “Expression of Cancer‐Testis Antigens MAGE‐A1, MAGE‐A4, NY‐ESO‐1 and PRAME in Bone and Soft Tissue Sarcomas: The Experience From a Single Center in China”

**DOI:** 10.1002/cam4.70971

**Published:** 2025-05-21

Chen A, Qiu Y, Yen YT, et al. Expression of Cancer‐Testis Antigens MAGE‐A1, MAGE‐A4, NY‐ESO‐1, and PRAME in Bone and Soft Tissue Sarcomas: The Experience From a Single Center in China. *Cancer Med*. 2025;14(7):e70750. https://doi.org/10.1002/cam4.70750

In the affiliation section of the manuscript, all of the affiliations were incorrect. The correct affiliations should be: “^1^Department of Oncology, Nanjing Drum Tower Hospital, Affiliated Hospital of Medical School, Nanjing University, Nanjing, China. ^2^The Comprehensive Cancer Center, Nanjing Drum Tower Hospital, Affiliated Hospital of Medical School, Nanjing University, Nanjing, China. ^3^Department of Pathology, Nanjing Drum Tower Hospital, Affiliated Hospital of Medical School, Nanjing University, Nanjing, China.”

We apologize for this error.

