# Supplementary material for: Expression of Cancer‐Testis Antigens MAGE‐A1, MAGE‐A4, NY‐ESO‐1 and PRAME in Bone and Soft Tissue Sarcomas: The Experience From a Single Center in China
Source: Cancer Med. 2025 Mar 28;14(7):e70750. doi: 10.1002/cam4.70750 (PMC11951172; doi:10.1002/cam4.70750)
Supplement: Supplementary file 1 — Table S1. [file CAM4-14-e70750-s001.docx]

| CTAs | MAGE-A1 | | | | MAGE-A4 | | | | NY-ESO-1 | | | | PRAME | | | | KK-LC | Total |
| --- | --- | --- | --- | --- | --- | --- | --- | --- | --- | --- | --- | --- | --- | --- | --- | --- | --- | --- |
|  | S | M | W | P | S | M | W | P | S | M | W | P | S | M | W | P | P | P |
| osteosarcoma | 8 | 3 | 2 | 13 | 4 | 3 | 9 | 16 | 0 | 1 | 0 | 1 | 2 | 2 | 2 | 6 | - | 36 |
| liposarcoma | 2 | 1 | 0 | 3 | 2 | 1 | 2 | 5 | 2 | 0 | 1 | 3 | 2 | 0 | 2 | 4 | - | 15 |
| leiomyosarcoma | 1 | 3 | 0 | 4 | 1 | 2 | 0 | 3 | - | - | - | - | 0 | 1 | 6 | 7 | - | 14 |
| UPS | 3 | 3 | 0 | 6 | 1 | 1 | 5 | 7 | 1 | 1 | 0 | 2 | 0 | 2 | 8 | 10 | - | 25 |
| chondrosarcoma | - | - | - | - | - | - | - | - | - | - | - | - | 0 | 0 | 1 | 1 | - | 1 |
| total | 14 | 10 | 2 | 26 | 8 | 7 | 16 | 31 | 3 | 2 | 1 | 6 | 4 | 5 | 19 | 28 | - | 91 |

**Extended table 1 Expression intensity and frequency of different kinds of sarcoma.** S, strong positive, +++/++++; M, medium positive, ++; W, weak positive, +; P, positive.

|  | 1 | 2 | 3 | 4 | P | N |
| --- | --- | --- | --- | --- | --- | --- |
| osteosarcoma | 12 | 6 | 2 | 1 | 22(55%) | 18(45%) |
| liposarcoma | 4 | 0 | 1 | 2 | 7(25%) | 21(75%) |
| leiomyosarcoma | 5 | 3 | 1 | 0 | 9(35%) | 17(65%) |
| UPS | 8 | 6 | 2 | 0 | 16(76%) | 5(24%) |
| chondrosarcoma | 1 | 0 | 0 | 0 | 1(8%) | 12(92%) |
| total | 30 | 15 | 6 | 3 | 55(43%) | 73(57%) |

**Extended table 2** The numbers of samples of different sarcomas expressing only one kind of CTA, two CTAs, three CTAs, four CTAs, or none of the CTAs.N, negative; P, positive.

| Diagnosis | Variables | | MAGE-A1 | | MAGE-A4 | | NY-ESO-1 | | PRAME | | Total | |
| --- | --- | --- | --- | --- | --- | --- | --- | --- | --- | --- | --- | --- |
|  |  |  |  |  |  |  |  |  |  |  |  |  |
|  |  |  | P | N | P | N | P | N | P | N | P | N |
| Osteosarcoma | Sex | M | 7 | 14 | 7 | 14 | 0 | 21 | 3 | 18 | 12 | 9 |
|  |  | F | 6 | 13 | 9 | 10 | 1 | 18 | 3 | 16 | 10 | 9 |
|  | Age | I | 4 | 9 | 6 | 7 | 0 | 13 | 4 | 9 | 8 | 5 |
|  |  | Y | 7 | 17 | 7 | 17 | 1 | 23 | 2 | 22 | 11 | 13 |
|  |  | O | 2 | 1 | 3 | 0 | 0 | 3 | 0 | 3 | 3 | 0 |
|  | Diameter | T1 | 2 | 5 | 1 | 6 | 0 | 7 | 0 | 7 | 2 | 5 |
|  |  | T2 | 4 | 9 | 6 | 7 | 0 | 13 | 2 | 11 | 8 | 5 |
|  |  | T3 | 3 | 10 | 6 | 7 | 0 | 13 | 2 | 11 | 7 | 6 |
|  |  | T4 | 3 | 2 | 1 | 4 | 1 | 4 | 2 | 3 | 3 | 2 |
| Liposarcoma | Sex | M | 3 | 8 | 4 | 7 | 3 | 8 | 2 | 9 | 4 | 7 |
|  |  | F | 0 | 17 | 1 | 16 | 0 | 17 | 2 | 15 | 3 | 14 |
|  | Age | I | - | - | - | - | - | - | - | - | - | - |
|  |  | Y | 1 | 21 | 3 | 19 | 1 | 21 | 2 | 20 | 5 | 17 |
|  |  | O | 2 | 4 | 2 | 4 | 2 | 4 | 2 | 4 | 2 | 4 |
|  | Diameter | T1 | 0 | 1 | 0 | 1 | 0 | 1 | 0 | 1 | 0 | 1 |
|  |  | T2 | 1 | 6 | 2 | 5 | 1 | 6 | 1 | 6 | 2 | 5 |
|  |  | T3 | 0 | 5 | 0 | 5 | 0 | 5 | 0 | 5 | 0 | 5 |
|  |  | T4 | 2 | 12 | 3 | 11 | 2 | 12 | 3 | 11 | 5 | 9 |
| Leiomyosarcoma | Sex | M | 1 | 3 | 0 | 4 | 0 | 4 | 1 | 3 | 2 | 2 |
|  |  | F | 3 | 19 | 3 | 19 | 0 | 22 | 6 | 16 | 7 | 15 |
|  | Age | I | - | - | - | - | - | - | - | - | - | - |
|  |  | Y | 3 | 16 | 2 | 17 | 0 | 19 | 5 | 14 | 6 | 13 |
|  |  | O | 1 | 6 | 1 | 6 | 0 | 7 | 2 | 5 | 3 | 4 |
|  | Diameter | T1 | 2 | 4 | 1 | 5 | 0 | 6 | 2 | 4 | 3 | 3 |
|  |  | T2 | 2 | 9 | 1 | 10 | 0 | 11 | 4 | 7 | 5 | 6 |
|  |  | T3 | 0 | 6 | 1 | 5 | 0 | 6 | 1 | 5 | 1 | 5 |
|  |  | T4 | 0 | 3 | 0 | 3 | 0 | 3 | 0 | 3 | 0 | 3 |
| UPS | Sex | M | 2 | 9 | 3 | 8 | 2 | 9 | 5 | 6 | 8 | 3 |
|  |  | F | 4 | 5 | 3 | 6 | 0 | 9 | 6 | 3 | 7 | 2 |
|  | Age | I | - | - | - | - | - | - | - | - | - | - |
|  |  | Y | 4 | 9 | 3 | 10 | 1 | 12 | 7 | 6 | 10 | 3 |
|  |  | O | 2 | 5 | 3 | 4 | 1 | 6 | 4 | 3 | 5 | 2 |
|  | Diameter | T1 | 2 | 7 | 3 | 6 | 0 | 9 | 5 | 4 | 6 | 3 |
|  |  | T2 | 3 | 6 | 3 | 6 | 1 | 8 | 6 | 3 | 7 | 2 |
|  |  | T3 | 1 | 0 | 0 | 1 | 0 | 1 | 0 | 1 | 1 | 0 |
|  |  | T4 | 0 | 1 | 0 | 1 | 0 | 1 | 1 | 0 | 1 | 0 |
| Chondrosarcoma | Sex | M | - | - | - | - | - | - | 0 | 8 | 0 | 8 |
|  |  | F | - | - | - | - | - | - | 1 | 3 | 1 | 3 |
|  | Age | I | - | - | - | - | - | - | - | - | - | - |
|  |  | Y | - | - | - | - | - | - | 1 | 8 | 1 | 8 |
|  |  | O | - | - | - | - | - | - | 0 | 3 | 0 | 3 |
|  | Diameter | T1 | - | - | - | - | - | - | 1 | 3 | 1 | 3 |
|  |  | T2 | - | - | - | - | - | - | 0 | 4 | 0 | 4 |
|  |  | T3 | - | - | - | - | - | - | 0 | 3 | 0 | 3 |
|  |  | T4 | - | - | - | - | - | - | 0 | 1 | 0 | 1 |

Extended table 3 Immunohistochemical results according to gender, age, and diameter. P, positive; N, negative; M, male; F, female; I, impubes, ＜18y; Y, ≥18y &＜ 65y; O, ≥65y; T1, ≤5 cm;T2, ＞5 cm&≤10 cm; T3, >10cm&≤15cm; T4, ＞15 cm.

|  | Sex | Age | Diameter （T1~4） | MAGE-A1 | MAGEA-4 | KK-LC-1 | NY-ESO-1 | PRAME |
| --- | --- | --- | --- | --- | --- | --- | --- | --- |
| osteosarcoma（n=40) | M | T | 1 | N | N | N | N | N |
|  | M | Y | 3 | N | S | N | N | N |
|  | F | Y | 1 | N | N | N | N | N |
|  | M | Y | 2 | N | N | N | N | N |
|  | M | T | 2 | N | W | N | N | N |
|  | F | Y | 2 | N | N | N | N | N |
|  | M | O | 2 | N | M | N | N | N |
|  | M | Y | 4 | M | N | N | N | N |
|  | M | Y | 3 | N | W | N | N | M |
|  | F | Y | 3 | N | N | N | N | N |
|  | M | T | 4 | N | N | N | N | N |
|  | F | Y | 1 | N | N | N | N | N |
|  | F | T | 3 | N | N | N | N | N |
|  | F | T | 2 | S | S | N | N | S |
|  | F | Y | 3 | W | W | N | N | N |
|  | M | T | 2 | N | W | N | N | N |
|  | F | Y | 3 | N | W | N | N | N |
|  | F | Y | 3 | N | N | N | N | N |
|  | F | Y | 2 | N | W | N | N | N |
|  | F | Y | 4 | S | S | N | M | M |
|  | F | Y | 1 | S | S | N | N | N |
|  | F | Y | 2 | N | N | N | N | N |
|  | F | T | 2 | S | N | N | N | W |
|  | M | T | 3 | N | N | N | N | N |
|  | M | T | 3 | M | M | N | N | W |
|  | M | T | 2 | N | N | N | N | N |
|  | F | T | N/A | N | W | N | N | N |
|  | M | O | N/A | S | W | N | N | N |
|  | M | Y | 1 | N | N | N | N | N |
|  | M | Y | 2 | N | N | N | N | N |
|  | M | Y | 2 | W | N | N | N | N |
|  | F | Y | 4 | N | N | N | N | N |
|  | M | Y | 1 | M | N | N | N | N |
|  | M | Y | 1 | N | N | N | N | N |
|  | M | Y | 3 | S | N | N | N | N |
|  | M | T | 4 | S | N | N | N | S |
|  | F | T | 3 | N | W | N | N | N |
|  | F | O | 2 | S | M | N | N | N |
|  | M | Y | 3 | N | N | N | N | N |
|  | F | Y | 3 | N | N | N | N | N |
| liposarcoma(n=28) | F | Y | 4 | N | N | N | N | N |
|  | F | Y | 4 | N | N | N | N | N |
|  | F | Y | 3 | N | N | N | N | N |
|  | M | Y | 2 | N | M | N | N | N |
|  | M | Y | 4 | N | N | N | N | N |
|  | F | Y | 2 | N | N | N | N | N |
|  | M | Y | 2 | N | N | N | N | N |
|  | F | Y | 4 | N | N | N | N | N |
|  | M | Y | N/A | N | N | N | N | N |
|  | M | Y | 3 | N | N | N | N | N |
|  | M | Y | 4 | M | W | N | W | N |
|  | M | Y | 3 | N | N | N | N | N |
|  | F | Y | 4 | N | W | N | N | N |
|  | F | Y | 4 | N | N | N | N | N |
|  | F | Y | 2 | N | N | N | N | N |
|  | F | Y | 4 | N | N | N | N | N |
|  | M | O | 4 | N | N | N | N | N |
|  | M | O | 2 | S | S | N | S | S |
|  | M | O | 4 | N | N | N | N | N |
|  | F | O | 3 | N | N | N | N | N |
|  | F | Y | 2 | N | N | N | N | N |
|  | F | Y | 4 | N | N | N | N | W |
|  | F | Y | 4 | N | N | N | N | W |
|  | F | Y | 2 | N | N | N | N | N |
|  | M | O | 4 | S | S | N | S | S |
|  | F | Y | 1 | N | N | N | N | N |
|  | F | Y | 4 | N | N | N | N | N |
|  | F | O | 3 | N | N | N | N | N |
| leiomyosarcoma(n=26) | F | Y | 3 | N | N | N | N | N |
|  | F | O | 2 | N | N | N | N | N |
|  | F | Y | 4 | N | N | N | N | N |
|  | F | Y | 1 | N | N | N | N | N |
|  | F | Y | 2 | N | N | N | N | N |
|  | F | O | 3 | N | N | N | N | N |
|  | F | O | 3 | N | M | N | N | W |
|  | F | O | 1 | N | N | N | N | N |
|  | M | O | 1 | M | N | N | N | N |
|  | F | Y | 1 | S | S | N | N | W |
|  | F | Y | 2 | N | N | N | N | M |
|  | F | Y | 1 | N | N | N | N | N |
|  | F | Y | 2 | M | N | N | N | W |
|  | F | Y | 3 | N | N | N | N | N |
|  | M | Y | 2 | N | N | N | N | N |
|  | F | O | 4 | N | N | N | N | N |
|  | F | Y | 2 | N | N | N | N | W |
|  | F | Y | 2 | N | N | N | N | N |
|  | F | Y | 1 | N | N | N | N | W |
|  | F | Y | 2 | N | N | N | N | N |
|  | M | O | 2 | N | N | N | N | W |
|  | F | Y | 2 | N | N | N | N | N |
|  | M | Y | 3 | N | N | N | N | N |
|  | F | Y | 3 | N | N | N | N | N |
|  | F | Y | 4 | N | N | N | N | N |
|  | F | Y | 2 | M | M | N | N | N |
| UPS(n=21) | F | Y | 1 | N | N | N | N | M |
|  | M | Y | 2 | N | N | N | N | W |
|  | M | Y | 4 | N | N | N | M | N |
|  | F | Y | 2 | S | N | N | N | W |
|  | M | O | 2 | N | N | N | S | W |
|  | M | O | 2 | S | S | N | N | M |
|  | M | Y | 2 | N | M | N | N | N |
|  | F | O | 1 | N | N | N | N | N |
|  | F | Y | 1 | M | N | N | N | W |
|  | M | O | 1 | N | N | N | N | N |
|  | M | O | 1 | S | W | N | N | N |
|  | F | Y | 1 | N | W | N | N | W |
|  | F | Y | 2 | N | N | N | N | N |
|  | F | O | 1 | N | W | N | N | W |
|  | M | Y | 1 | N | N | N | N | W |
|  | N/A | N/A | N/A | N | W | N | N | N |
|  | M | O | 2 | N | N | N | N | W |
|  | F | Y | 2 | M | W | N | N | W |
|  | M | Y | 2 | N | N | N | N | N |
|  | M | Y | 1 | N | N | N | N | N |
|  | F | Y | 3 | M | N | N | N | N |
| chondrosarcoma(n=13) | M | O | 3 | N | N | N | N | N |
|  | M | Y | 4 | N | N | N | N | N |
|  | F | Y | 1 | N | N | N | N | N |
|  | M | Y | 1 | N | N | N | N | N |
|  | M | Y | 1 | N | N | N | N | N |
|  | M | O | 2 | N | N | N | N | N |
|  | M | Y | 3 | N | N | N | N | N |
|  | F | Y | 1 | N | N | N | N | W |
|  | F | Y | 2 | N | N | N | N | N |
|  | F | Y | 2 | N | N | N | N | N |
|  | M | O | 2 | N | N | N | N | N |
|  | N/A | N/A | N/A | N | N | N | N | N |
|  | M | Y | 3 | N | N | N | N | N |

**Extended table 4** Detailed immunohistochemical results according to gender, age, and diameter. P, positive; N, negative; M, male; F, female; I, impubes, ＜18y; Y, ≥18y &＜ 65y; O, ≥65y; T1, ≤5 cm;T2, ＞5 cm&≤10 cm; T3, >10cm&≤15cm; T4, ＞15 cm.

| Reference | Region | Diagnosis | Sample size | Technology | Notes |
| --- | --- | --- | --- | --- | --- |
| [42] | Japan | Osteosarcoma | 16 | IHC | - |
|  |  | Liposarcoma | 93 |  |  |
|  |  | Chondrosarcoma | 14 |  |  |
| [43] | Japan | Osteosarcoma | 9 | IHC | - |
| [44] | Netherlands | Osteosarcoma | 9 | IHC&qPCR | Children |
| [45] | China | Osteosarcoma | 36 | IHC&PCR | Fresh tissue |
| [40] | USA | Liposarcoma | 37 | IHC&qPCR |  |
| [46] | Japan | Liposarcoma | 9 | IHC |  |
|  |  | Leiomyosarcoma | 11 |  |  |
|  |  | UPS | 23 |  |  |
| [47] | USA | Liposarcoma | 38 | IHC |  |
| [34] | USA | Liposarcoma | 25 | IHC |  |
| [49] | Japan & Canada & USA | Liposarcoma | 158 | Tissue Microarrays & IHC |  |
|  |  | Chondrosarcoma | 142 |  |  |
| [50] | Japan | Liposarcoma | 93 | IHC & WB &PCR |  |
| [52] | USA | Leiomyosarcoma | 24 | IHC |  |
| [53] | Germany | Leiomyosarcoma | 50 | IHC |  |
|  |  | UPS | 82 |  |  |
| [54] | Japan | UPS | 10 | IHC |  |
| [55] | USA | Chondrosarcoma | 11 | PCR |  |

**Extended table 5 Regions, technologies and sample sizes of the referenced studies.**
